# Resistance to *Nilaparvata lugens* in rice lines introgressed with the resistance genes *Bph14* and *Bph15* and related resistance types

**DOI:** 10.1371/journal.pone.0198630

**Published:** 2018-06-01

**Authors:** Yongqiang Han, Chao Wu, Lang Yang, Deyong Zhang, Yutao Xiao

**Affiliations:** 1 Agricultural Genomics Institute at Shenzhen, Chinese Academy of Agricultural Sciences, Shenzhen, China; 2 Institute of Plant Protection, Hunan Academy of Agricultural Sciences, Changsha, China; Fujian Agriculture and Forestry University, CHINA

## Abstract

Crop resistance is a cost-effective and environmentally friendly strategy for pest management. The brown planthopper (BPH, *Nilaparvata lugens*) is a devastating rice insect pest due to its ability to rapidly overcome plant resistance and the lack of sufficient resistance resources. BR4831 (a rice breeding line derived from the pyramiding of two BPH resistance genes, *Bph14* and *Bph15*, into the elite rice variety Huang-Hua-Zhan, HHZ) and two single-gene introgression lines (HF106, carrying *Bph14*, and C602, carrying *Bph15*, in the elite rice cultivar 9311) were evaluated for their resistance to BPH using a standard seed box screening test coupled with field tests. The related resistance types were determined using laboratory assays. The seed box test and laboratory biological assays showed that BR4831 exhibited strong antibiotic resistance, and the behavioral assay showed that this line also exhibited strong antixenotic resistance, while both HF106 and C602 exhibited only weak antibiosis and no antixenotic resistance. Field tests showed significantly improved resistance in BR4831 compared to that of its recipient parent HHZ and slightly increased resistance in HF106 and C602 in comparison with their recipient parent 9311. These results demonstrate that the rice line BR4831, with pyramided resistance genes, exhibits higher resistance than the monogenic lines HF106 and C602 and highlight the benefits of combining the seed box seedling test, field tests and laboratory assays to thoroughly analyze plant resistance types.

## Introduction

Rice (*Oryza sativa* L.) is a staple food for over one-third of the world’s population, but serious yield losses occur due to diseases and insect pests [1]. The brown planthopper (BPH) (*Nilaparvata lugens* Stål), one of the most devastating insect pests [2], is a monophagous vascular feeder that directly damages rice plants by sucking phloem sap from the rice leaf sheath and indirectly damages plants by transmitting plant viruses [2]. Chemical insecticides are extensively used for the control of BPH, which has resulted in many problems, including toxicity to natural enemies, pest resurgence [3], increased production cost, and long-term agro-ecosystem and human health damage [4].

Rice resistance is a cost-effective and environmentally friendly strategy for BPH management. To date, 32 BPH resistance genes have been identified in *indica* rice cultivars and related wild species [5]. However, few BPH-resistant rice varieties are widely cultivated [6] due to the ability of BPH to rapidly overcome plant resistance and a lack of sufficient resistance resources. Therefore, identifying new BPH-resistant germplasms and determining the associated resistance types are continuously needed.

Plant resistance to insect pests is categorized into three types: (1) antibiosis, which reduces herbivore performance following the ingestion of host tissue; (2) tolerance, in which herbivorous infestation is endured; and (3) antixenosis, which repels or disturbs insects, causing a reduction in colonization or oviposition [7]. Identifying these resistance types can help elucidate the mechanisms underlying resistance.

Two dominant resistance genes, *Bph14* and *Bph15*, identified in the B5 introgression line, were found to confer strong resistance to BPH [8]. *Bph14* is located on the long arm of chromosome 3, and *Bph15* is located on the short arm of chromosome 4 [8]. Using a map-based cloning approach, *Bph14* was cloned and was shown to encode a coiled-coil, nucleotide-binding, leucine-rich repeat (CC-NB-LRR) protein and to carry a unique LRR domain that might function in both recognizing BPH invasions and activating defense responses [9]. Recently, *Bph15* was accurately remapped to an approximately 580 kb genomic segment of B5 [10]. In previous studies, *Bph14*, *Bph15* and other resistance genes have been individually or pyramid-transferred into *indica* rice cultivars [11–19] using marker-assisted selection (MAS) to obtain rice lines with improved resistance. These improved rice lines showed higher resistance levels than their corresponding recipient parents. Moreover, the pyramid effect of multiple genes has been detected in BPH resistance. These results provide valuable information for the proper utilization of BPH resistance genes.

In the last decade, several elite rice varieties have been bred and commercially planted in China. One such variety is 9311, which is widely planted throughout China. Hybrids from the 9311 parent have accounted for most of the rice cultivation in China in the past 10 years [13]. Huang-Hua-Zhan (HHZ) is another widely planted *indica* rice variety in southern China that has a high yield and shows good quality [20]. However, these varieties are susceptible to BPH due to the absence of BPH resistance genes [13]. In a recent endeavor to breed new rice lines with B5 as the donor parent and HHZ and 9311 as the recipient parents, homozygous stable lines were selected from the BC_3_F_4_ generation in combination with MAS. Although these stable rice lines are expected to show BPH resistance, this has yet to be demonstrated. For the potential incorporation of these lines in further rice breeding, the levels of BPH resistance in the rice lines HF106, C602 and BR4831 and their parents (B5, 9311 and HHZ) were evaluated in both semifield and field conditions. The resistance types of these lines were assessed in the laboratory using a series of biological and behavioral assays. The present study is of significance for future BPH resistance evaluations and for the further breeding of rice varieties to achieve high resistance to BPH.

## Materials and methods

### BPH population and rice germplasms

BPH originated from samples collected from paddy fields at the Scientific Observing and Experimental Station of Crop Pests in Guilin, Ministry of Agriculture, China (25°36′00″N and 110°41′24″E). The insects were reared for approximately 10 generations on potted rice seedlings (var. TN1) in insect-proof cages. These insects were used in the tests unless otherwise indicated.

The rice lines HF106 (*Bph14*), C602 (*Bph15*) (BC_3_F_4_, 9311 × B5), and BR4831 (BC_3_F_4_, HHZ × B5, *Bph14* and *Bph15*), as well as their corresponding parents B5, 9311 and HHZ, were kindly provided by Wuhan University and the Hunan Academy of Agricultural Sciences and used in the tests. A BPH-resistant variety, IR64, and a BPH-susceptible variety, TN1, were included in the tests as controls. These two varieties were kindly supplied by the China National Rice Research Institute. Hereafter, the rice lines and varieties are collectively referred to as germplasms.

### Assessment of resistance at the rice seedling stage

We used the standard seed box screening test to evaluate BPH resistance in the rice germplasms. This test method is widely used in rice breeding programs and has been described in detail in several publications [7]. The seed box test is a choice test by which rice germplasms can be ranked according to damage caused by planthopper nymphs.

Twenty germinated seeds of each germplasm were evenly seeded at a 0.5 cm depth within a row in a seed box (L × W × H, 75 × 45 × 15 cm), which was filled with paddy soil to a depth of 3–4 cm, and were then covered with a thin layer of fine dry soil, which was subsequently wetted. The seeding rows in the box were 5 cm apart and were arranged in a randomized design with respect to the different germplasms in a box. At the edges of the box, TN1 seeds were sown in two rows to provide protection. After seeding, the box was placed in an 80-mesh insect-proof cage (L × W × H, 95 × 95 × 95 cm). The emerged seedlings were allowed to develop for 7 days and then thinned to 18 plants per row, and any weeds in the box were removed.

To obtain BPH nymphs for the test, potted TN1 tillering plants were exposed to 200 gravid females for oviposition in a cage. The potted plants were replaced every 24 h, and the replaced seedlings were transferred to different cages according to the exposure date. The newly hatched nymphs were allowed to develop to the second/third instar when used for the test.

At the seedling two-leaf stage in the seed boxes, potted plants with second-/third-instar BPH nymphs were gently transported to the boxes and patted to drop the nymphs onto the seedlings. During the process of slowly moving the potted plants over the boxes, the dropped nymphs were visually estimated to drop approximately 8–10 nymphs onto each seedling. Thereafter, the boxes were returned the cages individually.

When the TN1 seedlings in a box had become completely wilted due to planthopper feeding, the tests were terminated, and the damage to all seedlings in a box was scored according to Horgan et al. [6] (Table 1), where higher scores indicated greater susceptibility to BPH. The tests were conducted in 10 replicates in a greenhouse at the experimental station.

**Table 1:** Evaluation standard for rice resistance to planthoppers based on seedling mortality (adapted from Horgan et al. 2015).

Score	Rice damage	Resistance level
0	No damage	Immune
1	Slight damage to a few plants within a row	Highly resistant
3	First and second leaves of each plant partially yellowing	Resistant
5	Pronounced yellowing or stunting of plants, or 10–25% of plants wilted within a row	Moderately resistant
7	More than 50% of plants wilted or dead and the remaining plants severely stunted or dying	Moderately susceptible
9	All plants wilted or dead	Susceptible

### Assessment of resistance at the adult rice stage

The resistance of the rice germplasms to BPH at the rice adult stage was also assessed in the fields of the experimental station from May to October 2017. Rice seedlings (30 days old) of the eight germplasms were separately transplanted to a 60 m^2^ plot (10 × 6 m) on June 20, 2017. The seedlings were spaced at 20 × 25 cm, and 3 plots were allocated to each germplasm, totaling 24 plots. The plots were arranged in a complete randomized design and separated by a 50-cm bank. The plants were exposed to a natural BPH infestation and were not sprayed for pest control. The watering and fertilization of the plots were managed conventionally.

Beginning 20 days after transplantation (DAT), the plots were scouted to determine the BPH population size 5 times. During each scouting event, a plot was sampled at 4 adjacent rice hills per point at 5 random points. The rice hills at a sampling point were encircled with an iron sheet cylinder (30 cm in diameter, 50 and 80 cm in height before and after the elongation stage, respectively), and arthropods within the cylinder were collected using a vacuum sucker. All the arthropods collected from a plot were pooled together, and the numbers of BPHs in the samples were recorded. The data were converted to the number per 100 rice hills.

### Laboratory bioassay for resistance categories

#### Culture of insect-free rice plants

Insect-free rice plants were used in the following tests at the Agricultural Genomics Institute at Shenzhen, Chinese Academy of Agricultural Sciences. Seedlings were cultured in plastic nursery plates in 80-mesh insect-proof cages (L × W × H, 95 × 95 × 95 cm). Twenty-day-old seedlings were transplanted at one single-plant hill per pot in 4 L plastic buckets or at three two-plant hills per pot in 6 L plastic buckets. The former group of potted plants was used to test the settling preference of adult BPH females, while the latter group of potted plants was used in all other laboratory tests. Immediately after transplantation, the potted rice plants were protected from any insect damage using insect-proof cylindrical PVC cages. Before use in the following tests, yellow leaves and sheaths were removed from the plants.

#### Performance of BPH

BPH performance was tested in an arena consisting of a glass tube with two open ends (4 cm in diameter, 12 cm in height) in a climate-controlled room at 27 ± 1°C, under 75–80% relative humidity (RH) and a 16:8 (L:D) h photoperiod. A potted rice plant (30–45 DAT) was run through a glass tube inserted (1 cm) into the potting soil. A cut-out sponge slice (4 cm in diameter, 2 cm in height) encircling the rice stem was fit to the lower end of the tube to secure the rice plant and prevent drowning of the test insects. After 5 first-instar nymphs (< 24 h) were transferred to the plant in a glass tube, the top opening of the tube was sealed with a sponge slice to prevent insect escape. Three tubes were installed in the same manner per pot, and 48 tubes were prepared for each germplasm. The insects in the glass tubes were observed daily to record the developmental stage and number of nymphs surviving until adult emergence. The emerged adults were sucked out and sorted daily according to sex. The adult weight was obtained (Q/OAFA electronic balance, Mettler Toledo Instrument CO., LTD, Shanghai, China) individually for 30 randomly selected newly emerged adult females and males (< 24 h). The developmental duration was calculated for the nymphs. Forty-eight tubes per rice germplasm were randomly divided into 3 replicates to calculate nymphal survival rates (number of emerged adults/number of first-instar nymphs) and adult sex ratios (number of female adults/number of male adults).

The performance of adults originating from the above tests were further compared using the test arena described above. One macropterous BPH female and three males (< 24 h) resulting from the above tests were transferred to a plant (30–45 DAT) originating in the glass tube in the climate-controlled room. One female served as a replicate, and 15 females were tested for each rice germplasm. The adults were observed daily for survival until death. Adult longevity was obtained from the emergence date and death date. Beginning on the third day from the beginning of the test, nymphs on the stem, if any, were counted and removed daily until no nymphs were observed for 4 continuous days. Then, the rice stem between the two sponge slices was dissected under a microscope to assess the number of unhatched BPH eggs. The fecundity (sum of the number of nymphs and the number of unhatched eggs) and egg hatching rate (number of nymphs divided by fecundity) of each female were calculated.

The developmental duration of eggs was assessed to calculate laboratory population parameters. The procedures were largely the same as those utilized to assess adult BPH performance, except three macropterous gravid BPH females were confined to one plant of each rice germplasm within a tube and then removed after 24 h. The egg developmental duration was calculated as the difference between the oviposition date and the hatching date obtained from daily observation.

The BPH population parameters were calculated using an SAS program [21] with the data obtained above, which included the intrinsic rate of natural increase (*r*_*m*_), finite capacity of increase (*λ*), net reproductive rate (*R*_*0*_), and mean generation time (*T*).

#### Amount of feeding by adult BPH females

BPH honeydew excretion is an indicator of the amount of feeding [22]. The Parafilm sachet method was used to collect honeydew excreted by BPH female adults [22]. One macropterous BPH female, starved for 3 h, was transferred onto a Parafilm sachet (3.5 × 3.5 cm) attached to the stem of a potted rice plant (30–45 DAT) 5 cm above the soil surface in a climate-controlled room. The female was allowed to feed ad libitum for 72 h and then carefully removed from the sachet using an aspirator. The weight difference between the sachet before and after the test equaled the amount of excreted honeydew. Thirty BPH female adults were tested for each rice germplasm.

#### Settling preference of adult BPH females

The preferences of adult BPH females for the rice germplasms were tested in cages. One potted rice plant (30–45 DAT) of each rice germplasm was arranged evenly in a circle in a cylindrical mesh cage (90 cm diameter). Eighty macropterous BPH females (1–3 days old, starved for 3 h) in a plastic cup (5 cm diameter, 6 cm height) were placed in the center of the circular cage and allowed to freely select the plants. The number of insects on each plant was checked at 24, 72 and 120 h after the insects were introduced into the cage. The observations were repeated 10 times, and the relative positions of the potted plants were rotated between repetitions. The settling rates of the insects on the plants of different germplasms were calculated as the number of insects settled on a plant divided by the total number of insects.

### Data analysis

All data were subjected to analysis of variance (ANOVA) using SPSS 16.0 (SPSS Inc, New York, USA). All percentage and ratio data were subjected to arcsine square root transformation prior to ANOVA. Error assumption/homogeneity was verified using Kolmogorov-Smirnov and Levene’s tests. When a significant effect was observed, the means were separated by Tukey’s test (*P* = 0.05). The data in the tables and figures were calculated from original data.

## Results

### Resistance to BPH at the rice seedling stage

**Fig 1:**
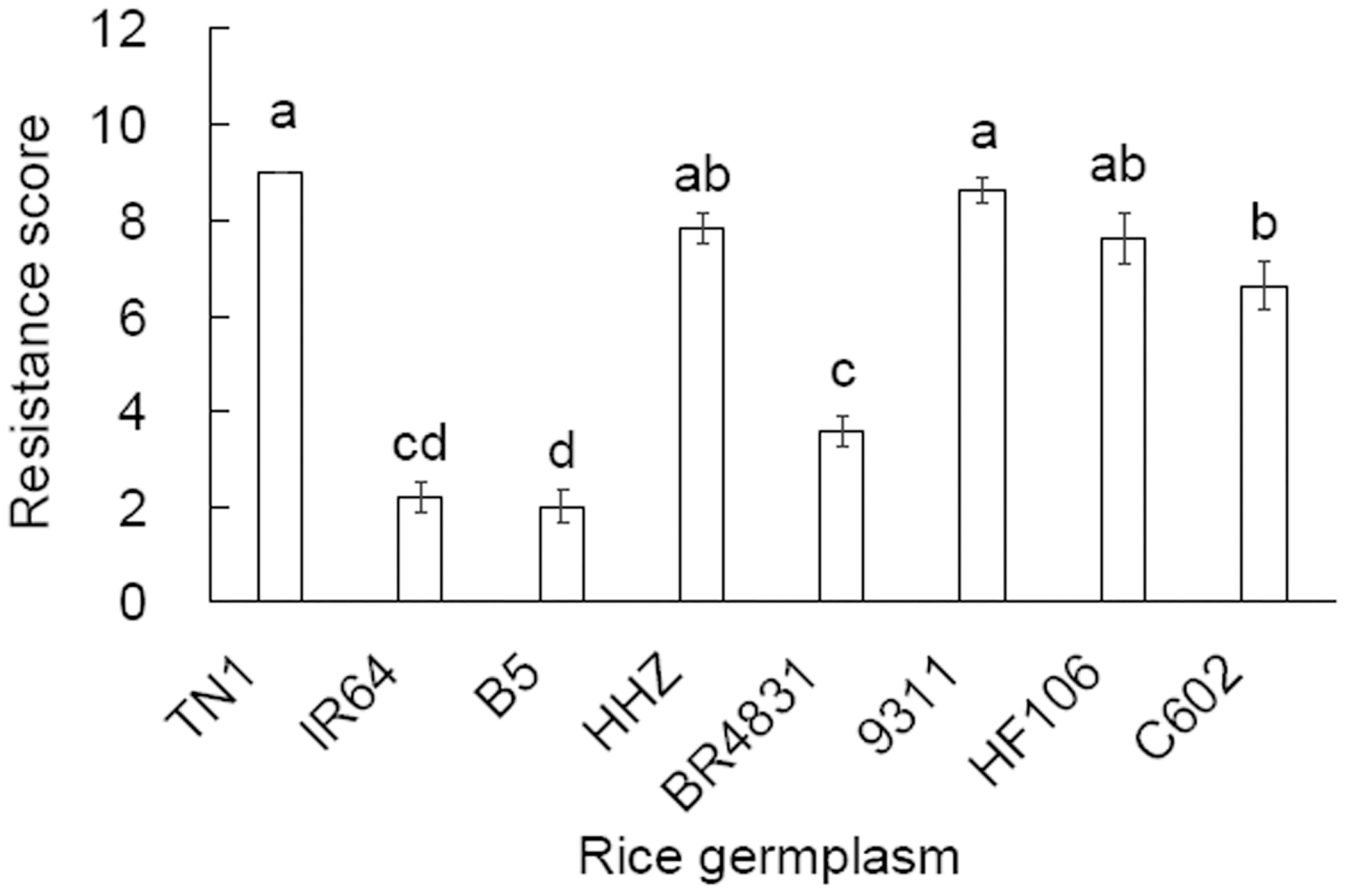
Resistance to *Nilaparvata lugens* assessed with the standard seed box test at the seedling stage of rice germplasms. Data are expressed as the mean ± SE from 10 replicates. Different letters over the bars indicate significant differences (Tukey HSD, *P* = 0.05).

In the standard seed box screening test, the seedlings of the rice germplasms showed significant differences in their resistance to BPH (*F* = 68.787, df = 7,79, *P* < 0.001; Fig 1). As expected, the rice varieties TN1, HHZ and 9311 were moderately susceptible or susceptible to BPH, while IR64 and B5 were highly resistant to BPH according to the evaluation standard of rice resistance to planthoppers (Table 1). BR4831, the sibling of B5 and HHZ that was pyramided with *Bph14* and *Bph15*, was resistant to BPH, scoring lower than HHZ and TN1. The siblings of B5 and 9311 were moderately susceptible or moderately resistant to BPH (C602, singly introgressed with *Bph15*) and moderately susceptible or susceptible to BPH (HF106, singly introgressed with *Bph14*).

### Resistance to BPH at the rice adult stage

BPH population sizes were monitored in field plots of the rice germplasms (Table 2). Significant differences were documented on all 5 monitoring dates (July 10, *F* = 4.552, df = 7,23, *P* = 0.006; July 25, *F* = 54.723, df = 7,23, *P* < 0.001; August 14, *F* = 19.692, df = 7,23, *P* < 0.001; August 29, *F* = 30.584, df = 7,23, *P* < 0.001; September 13, *F* = 10.363, df = 7,23, *P* < 0.001). As anticipated, on all 5 monitoring dates, the BPH populations between the IR64 and B5 plots and among the TN1, HHZ and 9311 plots were not significantly different (except on August 29 between TN1 and 9311). The BR4831 plots harbored significantly fewer BPH than TN1, except on July 10, and fewer BPH than HHZ (the recipient parent), except on July 10 and September 13. The BPH numbers observed between the BR4831 plots and the IR64 or B5 (the donor parent) plots differed only on July 25. The BPH population sizes in the HF106 and C602 plots and the TN1 and 9311 (the recipient parent) plots did not differ on any of the monitoring dates, except between C602 and 9311 on August 14.

**Table 2:** Population size (no./100 hills, mean ± SE) of *Nilaparvata lugens* in field plots (n = 3) of the TN1, IR64, B5, HHZ, BR4831, 9311, HF106, and C602 rice germplasms.

Rice germplasm	Monitoring date				
	July 10	July 25	August 14	August 29	September 13
TN1	158.3 ± 33.5 a	576.7 ± 38.4 ab	1361.7 ± 100.4 ab	918.3 ± 88.1 a	1223.3 ± 211.8 a
IR64	28.3 ± 10.1 c	93.3 ± 14.5 d	338.3 ± 15.9 d	138.3 ± 33.2 c	216.7 ± 16.9 c
B5	36.7 ± 14.2 bc	78.3 ± 15.9 d	361.7 ± 53.4 d	143.3 ± 23.5 c	251.7 ± 33.5 c
HHZ	138.3 ± 27.3 ab	551.7 ± 28.5 ab	1191.7 ± 119.6 ab	761.7 ± 60.9 ab	925.0 ± 108.5 ab
BR4831	116.7 ± 20.5 abc	276.7 ± 31.1 c	616.7 ± 79.1 cd	351.7 ± 33.8 c	536.7 ± 74.2 bc
9311	131.7 ± 27.3 abc	635.0 ± 34.0 a	1493.3 ± 154.7 a	641.7 ± 57.8 b	1151.7 ± 208.5 a
HF106	86.7 ± 19.6 abc	496.7 ± 25.2 ab	1013.3 ± 140.0 abc	806.7 ± 65.6 ab	658.3 ± 21.9 abc
C602	120.0 ± 16.1 abc	468.3 ± 38.8 b	923.3 ± 31.1 bc	658.3 ± 44.9 ab	861.7 ± 63.9 ab

Different letters following the means in a column indicate significant differences (Tukey HSD, *P* = 0.05).

### BPH performance

**Fig 2:**
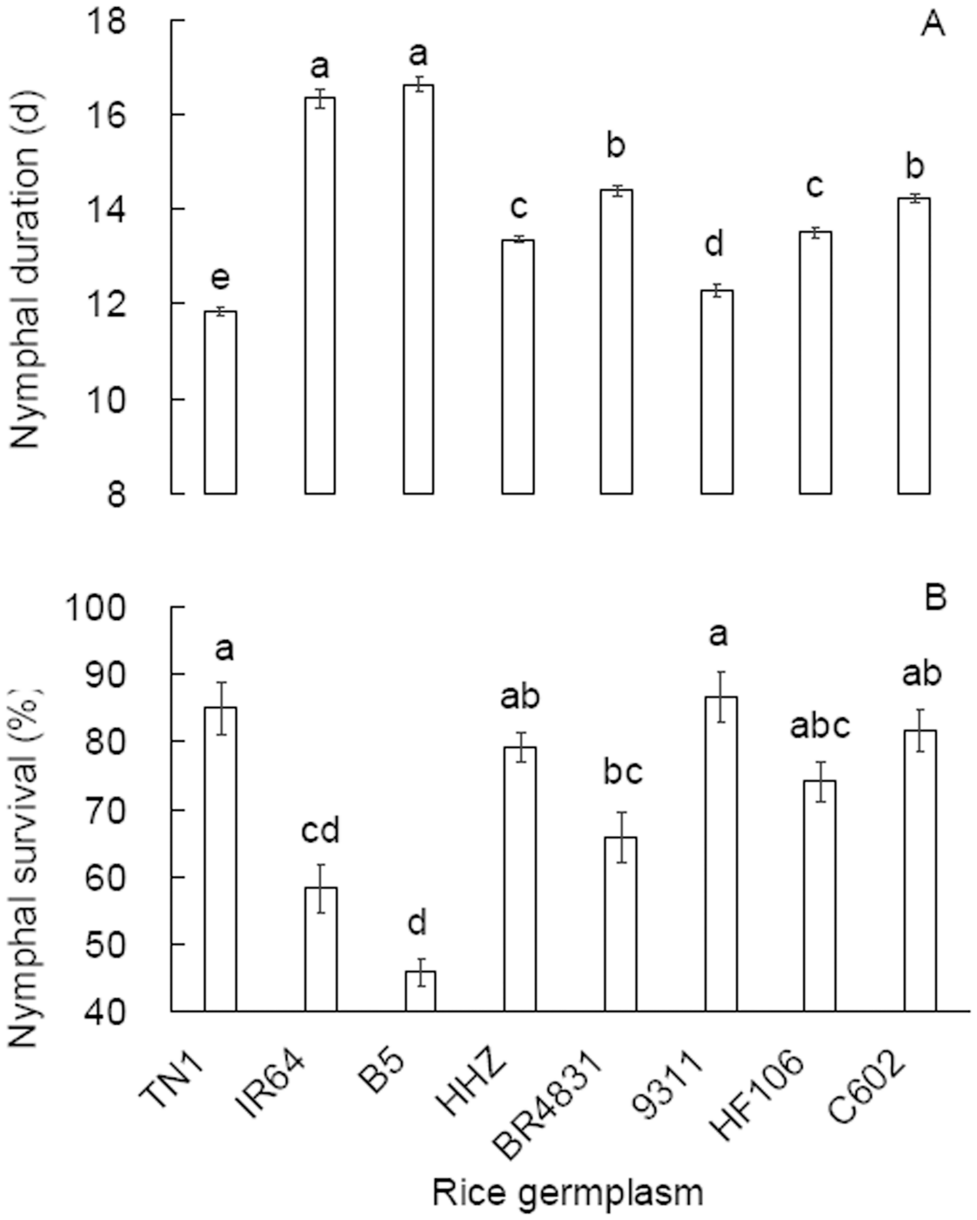
Developmental duration (A, n = 240) and survival (B, n = 3) (mean ± SE) of *Nilaparvata lugens* nymphs on rice germplasms. Different letters over the bars in a panel indicate significant differences (Tukey HSD, *P* = 0.05).

Under the no-choice tests, BPH nymphs exhibited different developmental durations on the rice germplasms (*F* = 425.038, df = 7,1383, *P* < 0.001; Fig 2A). Nymphal development was shorter on BR4831 and C602 than on B5 and IR64 but was retarded compared with that on the other rice germplasms. Nymphal survival also differed significantly between the rice germplasms (*F* = 16.535, df = 7,23, *P* < 0.001; Fig 2B). Fewer nymphs survived on BR4831 plants than on TN1 plants, and their survival rate was relatively low compared with that on HHZ plants. On C602 and HF106 plants, the nymphal survival rates decreased only marginally relative to those on TN1 and 9311 plants.

**Fig 3:**
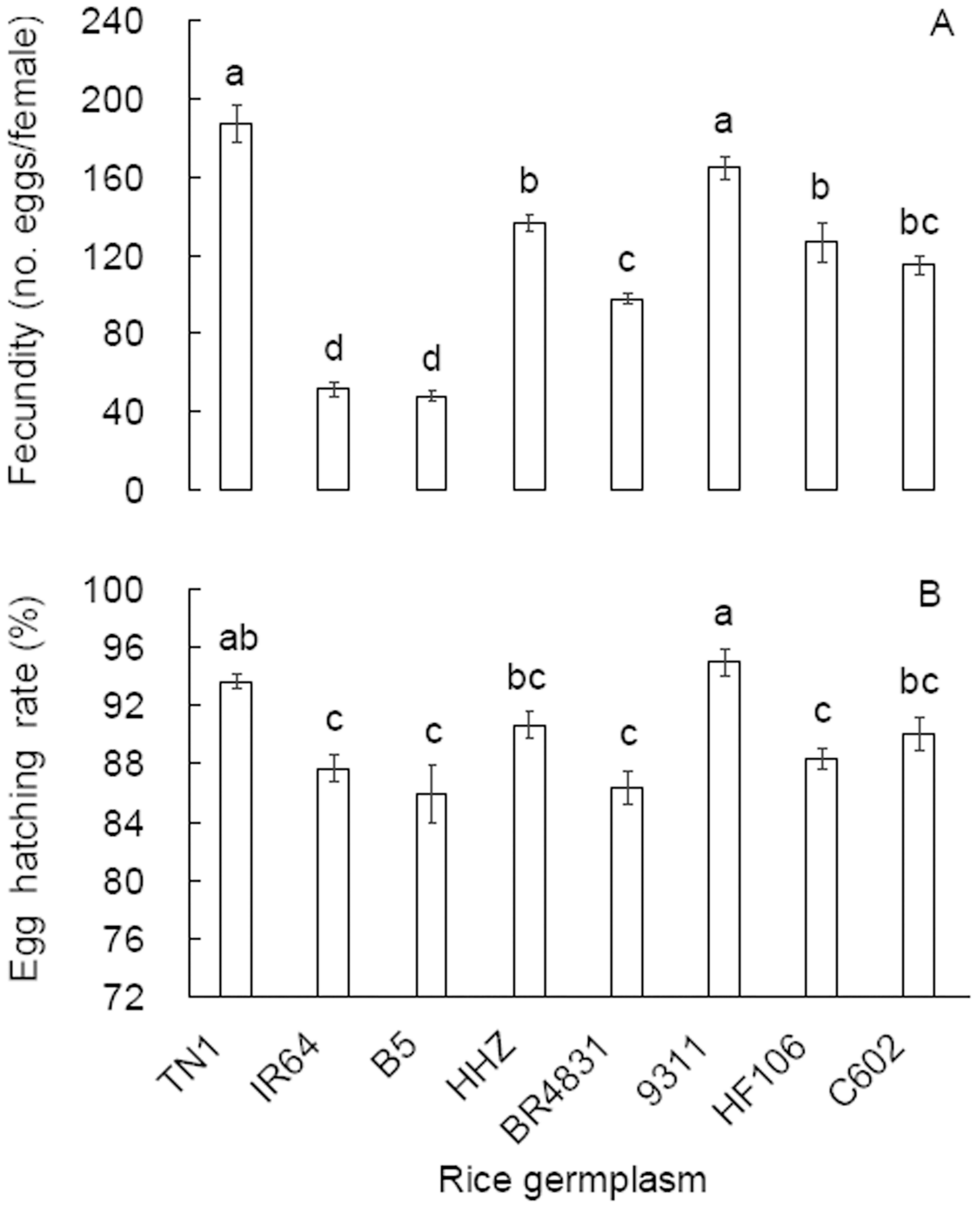
Fecundity (A) and egg hatchability (B) (mean ± SE, n = 15) of *Nilaparvata lugens* on rice germplasms. Different letters over the bars in a panel indicate significant differences (Tukey HSD, *P* = 0.05).

The fecundity of BPH females was significantly influenced by the rice germplasms on which the insects developed (*F* = 65.377, df = 7,119, *P* < 0.001; Fig 3A). BR4831 females oviposited fewer eggs than TN1 and HHZ females. Fewer eggs were also recorded from C602 and HF106 females than from TN1 and 9311 females. Eggs oviposited by females originating from the rice germplasms hatched at significantly different rates (*F* = 9.54, df = 7,119, *P* < 0.001; Fig 3B). The egg hatching rates of BR4831 and HF106 females did not differ from those of IR64 and B5 females but were lower than those of TN1 and 9311 females.

**Fig 4:**
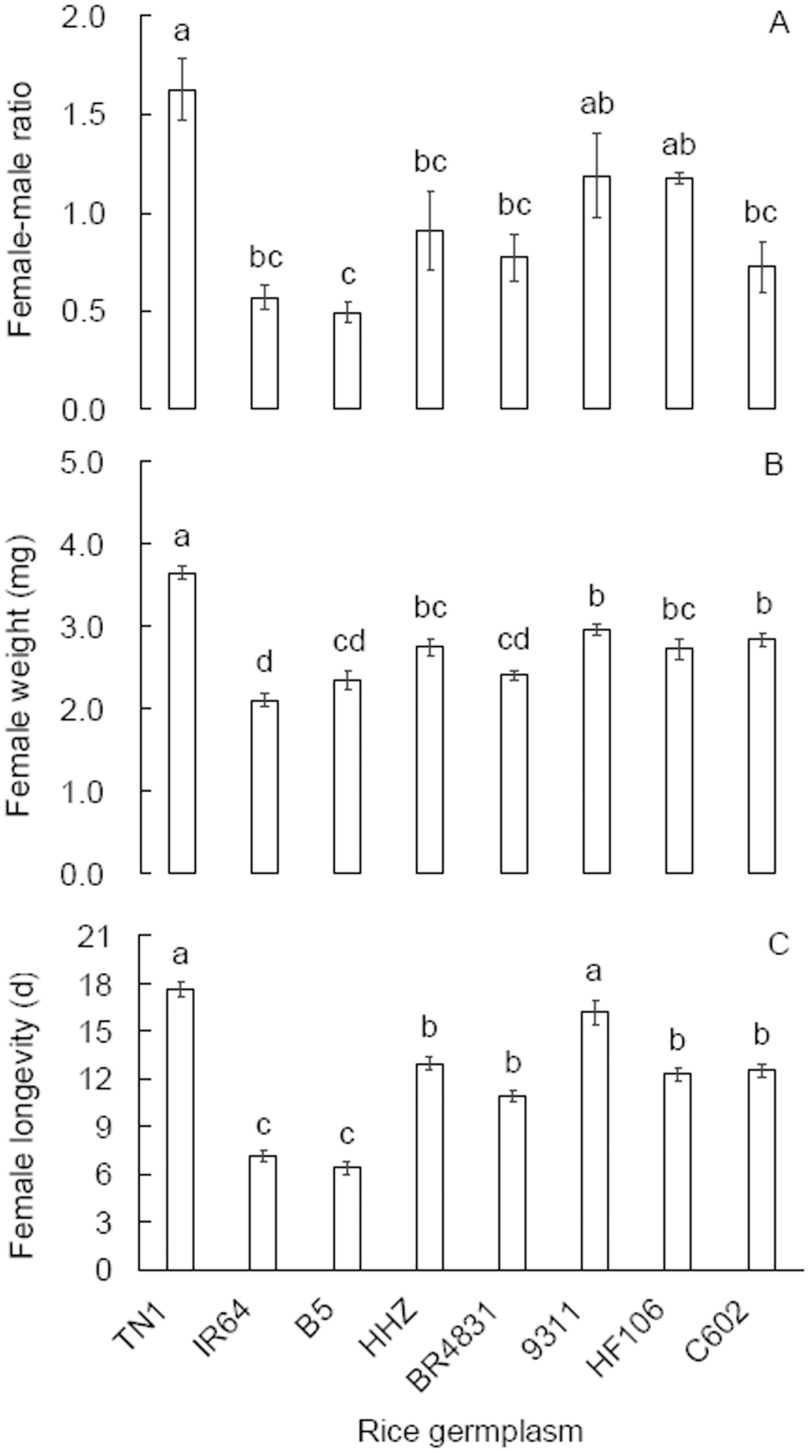
Sex ratio, female weight and longevity (mean ± SE) of *Nilaparvata lugens* developed on rice germplasms. A: Sex ratio (n = 3). B: Female weight (n = 30). C: Female longevity (n = 15). The different letters over the bars in a panel indicate significant differences (Tukey HSD, *P* = 0.05).

Adults derived from the rice germplasms exhibited different sex ratios (female/male) (*F* = 7.602, df = 7,23, *P* < 0.001; Fig 4A). The sex ratios of BR4831 and C602 adults were lower than those of TN1 adults and did not differ from those of IR64 and B5 adults. The female adults showed significantly different weights (*F* = 26.061, df = 7,239, *P* < 0.001, Fig 4B). The BR4831, HF106 and C602 females weighed less than the TN1 females (Fig 4B). The female adults also differed in their longevity (*F* = 65.905, df = 7,119, *P* < 0.001, Fig 4C). The BR4831, HF106 and C602 females displayed a significantly shorter lifespan than the TN1 females.

The BPH population parameters were significantly influenced by the rice germplasms (intrinsic rate of increase *r*_*m*_, *F* = 30.438, df = 7,23, *P* < 0.001; finite rate of increase *λ*, *F* = 29.937, df = 7,23, *P* < 0.001; net reproductive rate *R*_*0*_, *F* = 15.688, df = 7,23, *P* < 0.001; mean generation time *T*, *F* = 5.738, df = 7,23, *P* = 0.002; Table 3). The BR4831 BPH population exhibited lower *r*_*m*_, *λ* and *R*_*0*_ values than did the TN1 population, and these values were slightly lower compared to those of the HHZ population. Both the HF106 and C602 populations showed a lower *R*_*0*_ value than the TN1 population and slightly lower *r*_*m*_, *λ* and *R*_*0*_ values compared to the 9311 population. The BR4831 and HF106 populations completed a generation in a shorter time than the TN1 population, while both the B5 and IR64 populations required a significantly longer time to double their population sizes than all other populations (Table 3).

**Table 3:** Population parameters (mean ± SE) of *Nilaparvata lugens* fed on the TN1, IR64, B5, HHZ, BR4831, 9311, HF106, and C602 rice germplasms.

Rice germplasm	*r*_*m*_	*λ*	*R*_*0*_	*T*
TN1	0.143 ± 0.008 a	1.154 ± 0.009 a	214.8 ± 41.73 a	36.8 ± 0.70 a
IR64	0.067 ± 0.008 d	1.069 ± 0.008 d	9.6 ± 2.12 c	33.0 ± 0.13 bc
B5	0.054 ± 0.005 d	1.056 ± 0.005 d	6.0 ± 1.13 c	32.6 ± 0.58 c
HHZ	0.125 ± 0.008 abc	1.134 ± 0.009 abc	73.4 ± 13.68 bc	34.1 ± 0.71 bc
BR4831	0.098 ± 0.005 c	1.103 ± 0.006 c	29.1 ± 4.60 c	34.1 ± 0.16 bc
9311	0.136 ± 0.003 ab	1.145 ± 0.004 ab	128.4 ± 20.68 ab	35.5 ± 0.50 ab
HF106	0.127 ± 0.004 abc	1.135 ± 0.005 abc	72.7 ± 5.44 bc	33.9 ± 0.57 bc
C602	0.109 ± 0.005 bc	1.116 ± 0.006 bc	48.5 ± 8.95 bc	35.3 ± 0.87 abc

Data in a column followed by different letters are significantly different (Tukey HSD, *P* = 0.05). The observations were made in triplicate for each rice germplasm. *r*_*m*_, intrinsic rate of increase (eggs per female per d); *λ*, finite rate of increase (population growth rate per d); *R*_*0*_, net reproductive rate (eggs per female); *T*, mean generation time (d).

### Amount of feeding by adult BPH females

**Fig 5:**
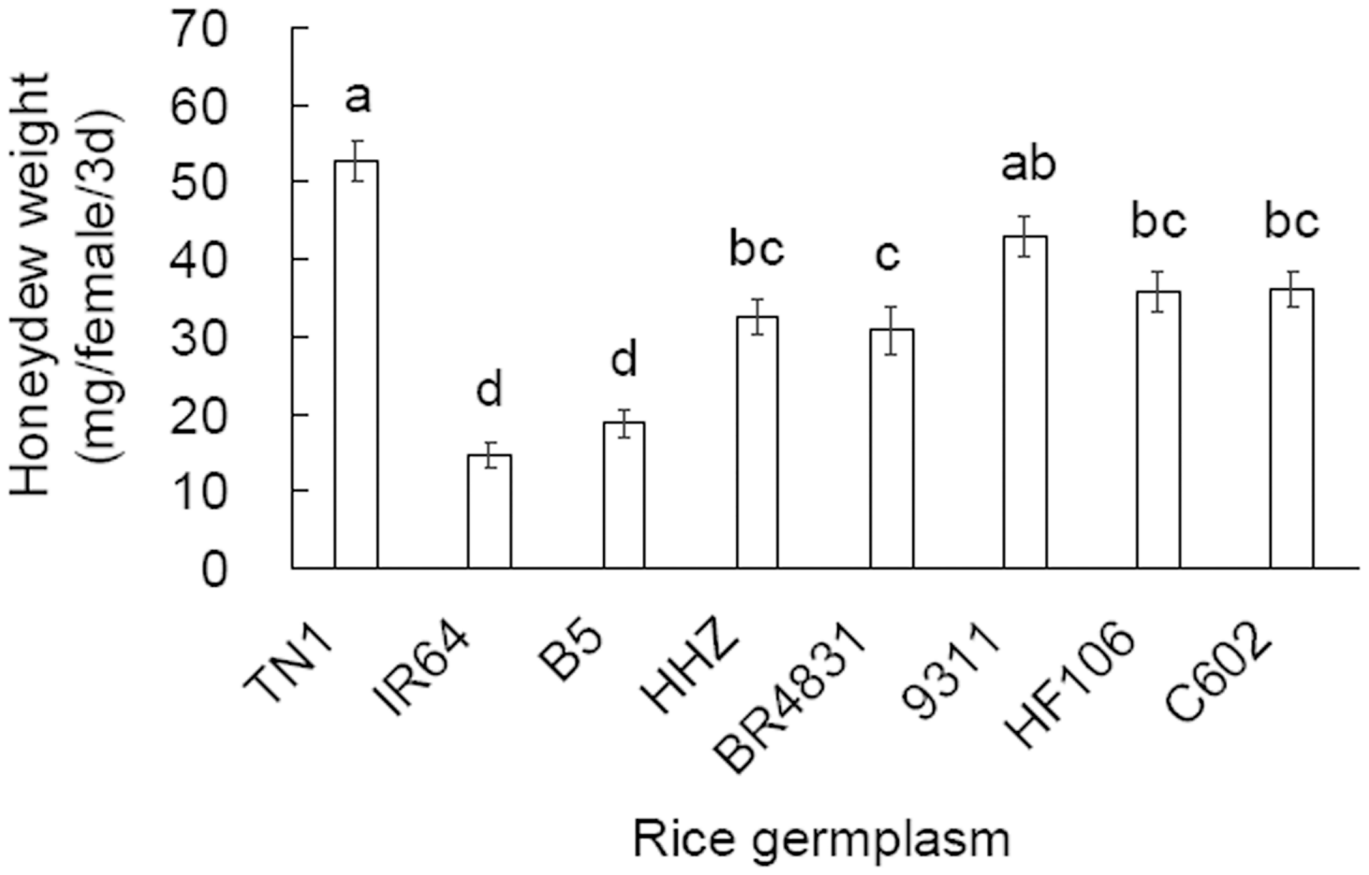
Weight of honeydew (mean ± SE, n = 30) excreted by female *Nilaparvata lugens* adults on rice germplasms. Different letters over the bars indicate significant differences (Tukey HSD, *P* = 0.05).

When confined in Parafilm sachets with plants of the rice germplasms, BPH females showed significantly different amounts of honeydew excretion (*F* = 25.39, df = 7,222, *P* < 0.001; Fig 5). The BPH females on the BR4831, HF106 and C602 plants excreted less honeydew than those on the TN1 plants, but more than those on their donor parent (B5) plants, while the honeydew excretions from these BPH females did not differ from those on their respective recipient parent plants.

### Female settling preference

**Fig 6:**
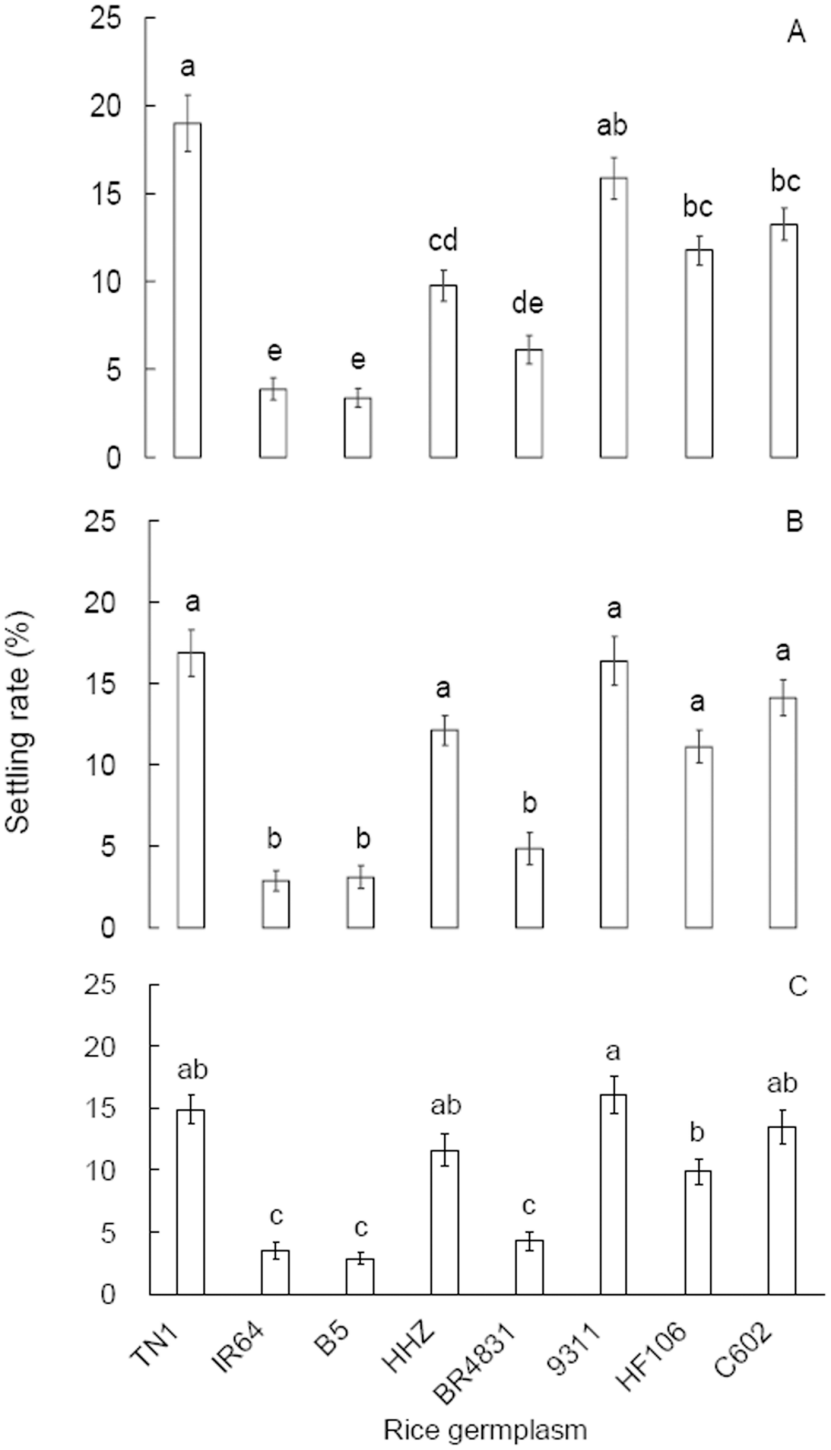
Settling preferences of macropterous female *Nilaparvata lugens* adults for plants of the rice germplasm at 24 h (A), 72 h (B) and 120 h (C) after insect release in the cage tests. Data are expressed as the mean ± SE from 10 replicates. Different letters over the bars in a panel indicate significant differences (Tukey HSD, *P* = 0.05).

In the cage tests, macropterous BPH females exhibited different settling preferences toward plants of the different rice germplasms at all 3 time points monitored (24 h: *F* = 38.025, df = 7,79, *P* < 0.001, Fig 6A; 72 h: *F* = 30.867, df = 7,79, *P* < 0.001, Fig 6B; 120 h: *F* = 29.343, df = 7,79, *P* < 0.001, Fig 6C). At all 3 time points, the females settled on B5, BR4831 and IR64 plants at similarly low rates. BR4831 deterred female settling compared with TN1 and HHZ, although the settling rates on BR4831 and HHZ plants did not differ at 24 h after insect release (Fig 6A). However, HF106 and C602 exerted no settling deterrence against BPH females compared with their recipient parent, 9311.

## Discussion

Crop resistance is a key component of integrated pest management because of its cost effectiveness and environmentally friendly nature. The migratory trait of BPHs makes precise control decisions difficult, resulting in frequent control spraying, even within a single rice season; therefore, incorporating crop resistance into the management of migratory BPHs is even more important than that for other insect pests. In an extensive evaluation of BPH resistance, Horgan et al. [6] found that only a few available resistance genes are effective in monogenic rice lines. Hence, thorough evaluation of rice resistance in the existing germplasms is critical for the identification and utilization of resistance genes against BPH [23].

Screening of rice plant resistance to planthoppers has been performed using a variety of methods, including field tests, mass screening in seed boxes, and modified seed box tests [23]. In the present investigation, we evaluated resistance levels in rice germplasms using a combination of these methods. The results of our seed box test indicated that the sibling rice line BR4831, from the B5 and HHZ parents, was resistant to BPH, and the sibling rice line C602, from the B5 and 9311 parents, was moderately susceptible or moderately resistant to BPH (Fig 1). Although the seed box test is a rapid and simple method to evaluate seedling susceptibility to planthoppers [6], this test assesses the responses of nymphs only under substantially simplified conditions. Our field tests confirmed the resistance level of BR4831 determined in the seed box tests, where the BPH population in the BR4831 plots was smaller than in the TN1 plots (the susceptible control) and did not differ from that in the IR64 plots (the resistant control) at 4 of the 5 monitoring times (Table 2). However, the field tests indicated that C602 was susceptible to BPH because the population size in the C602 plots did not differ from that in the TN1 plots at all 5 monitoring times (Table 2). Therefore, a field test over an entire season in combination with the seed box test is more appropriate for evaluating resistance levels in an array of plant germplasms.

Plant resistance to herbivores is classified as antibiosis, antixenosis or tolerance, and laboratory assays are necessary to differentiate the resistance types. In a no-choice design, we observed a significantly prolonged developmental duration and reduced survival of BPH nymphs on BR4831 plants than on TN1 (Fig 2), indicating that the rice line BR4831 is antibiotic to BPH nymphs. Furthermore, we recorded reduced fecundity, egg hatching rates, female ratios, female weights and longevity on BR4831 plants compared with TN1 plants and, to a lesser extent, on C602 and HF106 plants compared with TN1 plants (Figs 3 and 4). Similar antibiotic effects on BPH performance in introgressed rice lines were found when MH63 was used as the recipient parent [12]. These laboratory population parameters resulted in significantly lower net reproductive rates (*R*_*0*_) in the BR4831, C602 and HF106 populations (ranked from low to high) compared with the TN1 population (Table 3). Therefore, BR4831 exhibits obvious antibiotic resistance, while C602 and HF106 exhibit weak antibiotic resistance to BPH. The antibiotic resistance level in these rice germplasms was positively associated with the amount of BPH feeding (Fig 5), which has been reported frequently [24–25]. These laboratory test results explain the resistance levels of the rice germplasms recorded in the seed box test limited to the detection of antibiotic resistance [6].

A behavioral preference test under choice conditions is usually employed for the detection of antixenotic resistance in crop lines. BR4831 shows antixenotic resistance (Fig 6), as indicated by the consistently lower settling rates of BPHs on BR4831 plants compared with TN1 plants at all 3 monitoring times and compared with HHZ plants at 2 of the 3 monitoring times. Furthermore, the antixenosis observed in BR4831 was as strong as that in both B5 and IR64 (Fig 6). These results confirm the field test results showing that no differences in BPH population size existed between the BR4831 plots and either the IR64 or B5 plots. The rice lines C602 and HF106 showed no antixenosis against BPH compared with TN1 and their parent 9311. This observation may explain the equal population sizes in the field plots of these two rice lines and the TN1 and 9311 varieties, although C602 and HF106 showed enhanced or marginally increased antibiosis in the seed box test and laboratory assays compared with TN1 and 9311. In introgression rice lines harboring *Bph14* or *Bph15* with MH63 as the recipient parent, antixenosis to BPH is observed [12]. The difference in antixenosis observed in these sibling germplasms may be due to the different genetic backgrounds of the recipient parents.

We recorded obviously increased antibiotic and antixenotic resistance in BR4831 relative to its recipient parent HHZ and enhanced or marginally increased antibiosis in C602 and HF106 relative to their recipient parent 9311. BR4831 is a dual-gene (*Bph14* and *Bph15*) homozygous stable line, while C602 and HF106 are single-gene (*Bph14* or *Bph15*) homozygous lines, and BR4831 showed higher resistance to BPH than HF106 and C602 in both the seed box and field tests. Employing seed box tests, several previous studies involving the *Bph14* and *Bph15* genes [11–16, 26] as well other genes [19, 27] have demonstrated similar results regarding pyramided rice lines showing stronger resistance than single-gene lines. The antibiosis recorded herein with the two single-gene introgression lines C602 and HF106 was not as strong as that reported by Li et al. [11], where 92.3% of *Bph14* single-introgression lines exhibited moderate resistance, and all of the *Bph15* single-introgression lines showed resistance or high resistance in the seed box test. In another report involving the *Bph14* and *Bph15* genes introgressed into the recipient parent MH63, BPH resistance in the single-gene rice lines was improved compared with that in the recipient MH63 [12]. The genetic backgrounds of recipient parents can complicate the final resistance levels observed in introgressed lines. In the present study, B5 carried the resistance genes *Bph14* and *Bph15*, while 9311 and HHZ did not carry these genes [12, 13], and HF106 and C602, sibling lines of B5 and 9311, were confirmed to carry *Bph14* and *Bph15*, respectively. However, HF106 and C602 showed only marginal or no differences from HHZ and 9311 in terms of the measured parameters. Similar low resistance in some lines singly introgressed with *Bph14* has also been previously reported [11]. It may be that the resistance genes *Bph14* and *Bph15* are either not expressed or are not fully expressed in HF106 and C602, as indicated in a previous study showing that BPH resistance genes with a partial dominant effect result in moderate resistance or the maintenance of susceptibility to BPH in different genetic backgrounds [28]. Furthermore, the number of backcrosses involved in obtaining such introgressed lines can also affect the genetic background derived from the resistant parent. The rice lines used in this study resulted from three backcrosses and four selfings. BPH biotypes can also influence the detected resistance levels in introgressed lines. There are four BPH biotypes, and different biotypes show variable adaptations to assorted resistance genes [6]. The BPH population that we used in our tests may have overcome the single resistance gene present in HF106 and C602.

Overall, we observed increased BPH resistance in the introgressed rice line BR4831 using a combination of seedling (seed box) and field tests. Furthermore, antibiotic resistance was elucidated using laboratory assays, and antixenotic resistance was revealed using BPH settling behavior tests. Most studies employ the seedling test only for resistance evaluation of improved rice lines, which may lead to incomplete conclusions. Our results indicate that the dual-gene introgression rice line BR4831 is resistant to BPH via antibiosis and antixenosis, while the two single-gene lines HF106 and C602 show only weak antibiosis and no antixenotic resistance. These results confirm the hypothesis of Horgan et al. [6] that the pyramiding of two or more genes with strong to weak resistance could improve the strength and durability of resistance. Furthermore, our study highlights the importance of incorporating seedling tests, field tests and laboratory assays in the evaluation of planthopper resistance in rice germplasms, or even in other insect pest-crop systems.
